# Adherence to anti-retroviral therapy, decisional conflicts, and health-related quality of life among treatment-naïve individuals living with HIV: a DEARS-J observational study

**DOI:** 10.1186/s40780-023-00277-y

**Published:** 2023-03-02

**Authors:** Yusuke Sekine, Takashi Kawaguchi, Yusuke Kunimoto, Junichi Masuda, Ayako Numata, Atsushi Hirano, Hiroki Yagura, Masashi Ishihara, Shinichi Hikasa, Mariko Tsukiji, Tempei Miyaji, Takuhiro Yamaguchi, Ei Kinai, Kagehiro Amano

**Affiliations:** 1 Department of Pharmacy, Tokyo Medical University Hospital, 6-7-1 Nishishinjuku, Shinjuku-ku, Tokyo, 160-0023 Japan; 2 Department of Practical Pharmacy, School of Pharmacy, Tokyo University of Pharmacy and Life Sciences, Tokyo, Japan; 3 Department of Pharmacy, Sapporo Medical University Hospital, Sapporo, Japan; 4 Department of Pharmacy, Center Hospital of the National Center for Global Health and Medicine, Tokyo, Japan; 5 Department of Pharmacy, National Hospital organization Nagoya Medical Center, Nagoya, Japan; 6 Department of Pharmacy, National Hospital Organization Osaka National Hospital, Osaka, Japan; 7 Department of Pharmacy, Gifu University Hospital, Gifu, Japan; 8 Department of Pharmacy, Hyogo Medical University Hospital, Hyogo, Japan; 9 Division of Pharmacy, Chiba University Hospital, Chiba, Japan; 10 Division of Biostatistics, Tohoku University Graduate School of Medicine, Sendai, Japan; 11 Department of Laboratory Medicine, Tokyo Medical University, Tokyo, Japan

**Keywords:** Treatment-naïve people living with HIV, Anti-retroviral therapy, Decisional conflict, Adherence, Health-related quality of life

## Abstract

**Background:**

Supporting people living with HIV using anti-retroviral therapy (ART) is important due to the requirement for strict medication adherence. To date, no data from longitudinal studies evaluating adherence by treatment-naïve people living with HIV are currently available. We investigated the adherence of treatment-naïve people living with HIV over time and examined the relationships among decisional conflicts, adherence, and health-related quality of life (HRQL).

**Methods:**

The survey items included adherence (visual analogue scale [VAS]), decisional conflict (decisional conflict scale [DCS]), and HRQL (Medical Outcomes Study HIV Health Survey [MOS-HIV]). The DCS and MOS-HIV scores and the VAS and MOS scores were collected electronically at the ART initiation time point and at 4-, 24-, and 48-week post-treatment time points.

**Results:**

A total of 215 participants were enrolled. The mean DCS score was 27.3 (SD, 0.9); 23.3% of participants were in the high-score and 36.7% in the low-score groups. The mean adherence rates at 4, 24, and 48 weeks were 99.2% (standard error [SE], 0.2), 98.4% (SE, 0.4), and 96.0% (SE, 1.2), respectively. The least-square means of the MOS-HIV for the DCS (high vs. low scores) were 64.4 vs. 69.2 for general health perceptions and 57.7 vs. 64.0 for HRQL, respectively.

**Conclusion:**

Adherence among treatment-naïve people living with HIV was maintained at a higher level, and HRQL tended to improve with ART. People with high levels of decisional conflict tended to have lower HRQL scores. Support for people living with HIV during ART initiation may be related to HRQL.

## Background

Anti-retroviral therapy (ART) improves the prognosis for human immunodeficiency virus (HIV) infection [1], but it requires long-term treatment and the maintenance of strict adherence to treatment. To achieve treatment success, 95% adherence is required for early ART [2]; however, recent studies have reported treatment success rates of 98.9% and 96% with adherence rates of 90% or more and 80–90%, respectively [3]. In a meta-analysis of people living with HIV who are 12 to 24 years of age, 50–60% in Western countries and more than 70% in Asia reported good adherence [4]. Although advances in ART have improved treatment success rates, adherence remains important to achieving the best outcomes [5]. Therefore, treatment-naïve people living with HIV experience high levels of decisional conflict and anxiety caused by medical uncertainties at the start of treatment [6]. Negative attitudes toward treatment and low literacy levels [7, 8] are factors associated with non-adherence; however, good communication with healthcare professionals [8, 9] is associated with good adherence. Some reports have suggested that decisional conflict can be reduced with support from healthcare professionals [10], but this has not been confirmed [11]. However, people living with HIV have a poorer health-related quality of life (HRQL) than the general population [12]. Previous studies have reported that ART improves the HRQL and that good adherence is associated with good HRQL [13].

To date, no data from longitudinal studies evaluating adherence by treatment-naïve people living with HIV have been obtained. We hypothesized that adherence to ART by treatment-naïve people living with HIV would be associated with decisional conflict, with decreased decisional conflict leading to increased adherence and better HRQL.

This study assessed longitudinal adherence by treatment-naïve people living with HIV and analyzed relationships among adherence, decisional conflicts, and HRQL.

## Methods

### Study design and participants

This was a multicenter, observational study of treatment-naïve people living with HIV who newly initiated ART at eight medical institutions in Japan. The enrolment period was from 1 to 2017 to 31 March 2019. The eligibility criteria were age 20 years or older and treatment-naïve people living with HIV who had newly initiated ART. Participants with severe mental illness, cognitive impairment that might affect the completion of the questionnaire, and native speakers of a language other than Japanese were excluded.

### Ethical consideration

This study was conducted in accordance with the Declaration of Helsinki and Ethical Guidelines for Medical and Health Research Involving Human Subjects (Ministry of Education, Culture, Sports, Science, and Technology and Ministry of Health, Labour, and Welfare, 2014). The approval and supervision of the ethical review committee of each institution were obtained, and all participants provided informed consent. This study is registered with University Hospital Medical Information Network-Clinical Trials Registry (UMIN-CTR) (number: 000030146).

### Outcomes and measurements

#### Demographics

Participant characteristics, including age, sex, weight, history of hepatitis B virus, history of hepatitis C virus, AIDS, serum creatinine level, ART regimen, and virological outcome, were collected from the medical records. The following formula for the Japanese population was used to calculate the estimated glomerular filtration rate (eGFR) (mL/min/1.73 m^2^): 194 × [age]^−0.287^ × [serum creatinine (mg/dL)]^−1.094^ × 0.739 (females).

#### Decisional conflict

Decisional conflict was assessed using the decisional conflict scale (DCS) [14]. The DCS consists of 16 items and uses a 5-point Likert scale. Scores were calculated using a scale of 0 to 100. According to the user manual, we calculated the total score and the scores for the following subscales: informed, values clarity, support, uncertainty, and effective decision [15]. Higher scores indicated a higher conflict state of the participants. A total score < 25 (low) was associated with easier decision-making, and a total score ≥ 37.5 (high) was associated with delayed or uncertain decision-making. The effect size for a clinically meaningful difference was estimated to range from 0.3 to 0.4.

#### Medication adherence

Medication adherence was assessed using the visual analogue scale (VAS). The VAS has been used for clinical research and routine practice as a simple method of assessing medication adherence by people with HIV and has been associated with treatment effectiveness [16–18]. The VAS question was as follows: “What percentage of your medications did you take in the past month?” If the VAS score was not 100%, then a selective format was used to investigate why the dose was not administered.

#### Health-related quality of life

The HRQL was measured using the Medical Outcomes Study HIV Health Survey (MOS-HIV), specifically for individuals with HIV [19]. The MOS-HIV is a 35-item questionnaire that assesses the following subscales: general health perceptions, pain, physical functioning, role functioning, social functioning, energy/fatigue, mental health, health distress, cognitive function, quality of life, and health transition. The scales of the MOS-HIV are scored as summed rating scale scores using a scale of 0 to 100; higher scores indicate better health.

#### Virological outcomes

The HIV-1 viral load and CD4 counts were collected as virological outcomes. Virological success was defined as an HIV-1 viral load < 50 copies/mL at 48 weeks, following the Food and Drug Administration snapshot algorithm.

#### Data collection

At the time of ART initiation, DCS and MOS-HIV scores were collected. Adherence rates and MOS-HIV scores were collected at 4, 24, and 48 weeks after initiation. Patient-reported outcome instruments were administered by an electronic patient-reported outcome system (Viedoc Me; Pharma Consulting Group, Uppsala, Sweden). Depending on the WiFi environment, each site either chose to use the ‘bring your own device’ method or provided devices to collect electronic patient-reported outcomes. Researchers examined individual patient backgrounds at ART initiation and virological outcomes at baseline and 4, 24, and 48 weeks. Collected data were entered into the Electronic Data Capture system (Viedoc; Pharma Consulting Group).

### Study cohorts and analysis

Baseline demographics of participants were summarized using descriptive analyses. The VAS scores for medication adherence and MOS-HIV scores were calculated as means and 95% confidence intervals (CIs). Descriptive statistics were used to summarize reasons for not using medication. Longitudinal changes (weeks 4, 24, and 48) and these trends from baseline in the MOS-HIV scores across groups that were used to classify the DCS score level (high, normal, low) at ART initiation were estimated by constructing a general linear model that included the baseline MOS-HIV score, history of AIDS, CD4, and timepoint as explanatory variables (repeated measurements). An unstructured covariance matrix was assumed, and robust standard errors (SEs) were calculated as parameter estimates. All analyses were conducted using Statistical Analysis System (version 9.4; SAS Institute Inc., Cary, NC). Tests were two-sided, and the statistical significance level was 5%. No correction for multiple tests was performed.

## Results

### Participants

**Fig. 1:**
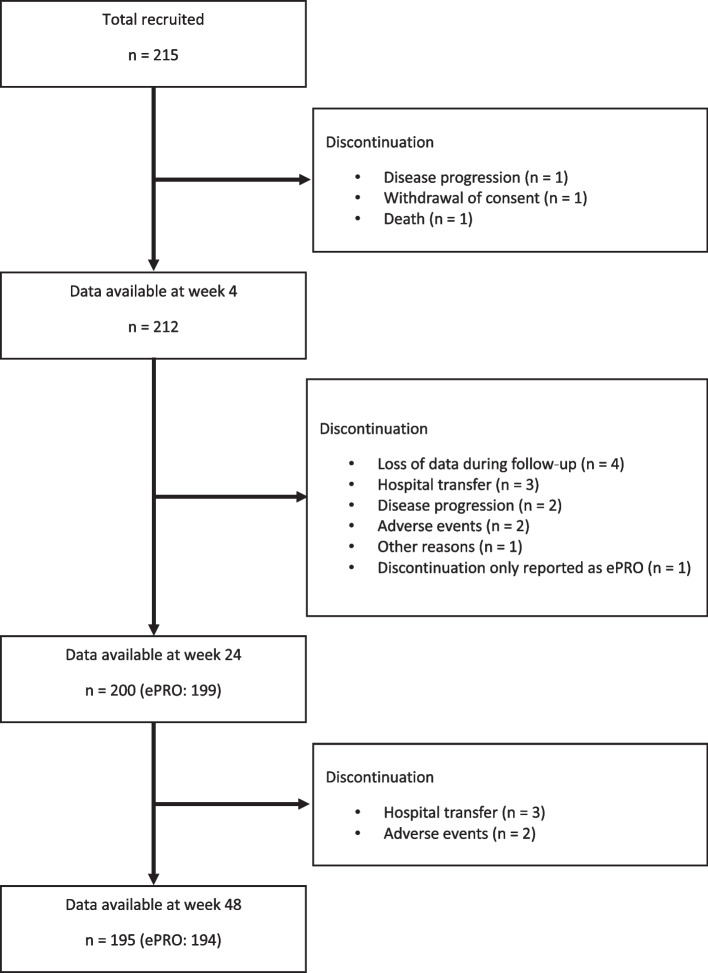
Participant flow chart. ePRO, electric patient-reported outcome

A total of 215 people living with HIV were recruited (Fig. 1). Baseline characteristics are summarized in Table 1. The mean age was 37.2 years (standard deviation (SD), 9.8 years), and 96.7% were males. Furthermore, 57.7% had hepatitis B virus-seronegative, 2.8% had an active hepatitis C virus infection, and 10.7% had AIDS. Most were prescribed two nucleoside reverse-transcriptase inhibitors and one integrase strand transfer inhibitor; 80% were prescribed a dolutegravir-based regimen and 35% were prescribed single-tablet regimens. At baseline, the median HIV-RNA viral load was 62,400 copies/mL (interquartile range (IQR), 18,500–151,500), the median CD4 + cell count was 314 cells/µL (IQR, 133–455.5 cells/µL), and the median estimated glomerular filtration rate was 90.9 mL/min/1.73 m^2^ (IQR, 78.2–102.5 mL/min/1.73 m^2^).

**Table 1:** Baseline characteristics (*n* = 215)

Characteristic		
Age, years, mean (SD)		37.2 (9.8)
Sex, n (%)		
Male		208 (96.7)
Female		7 (3.3)
Weight, kg, mean (SD)		65.2 (12.1)
HBV, n (%)		
Seronegative		124 (57.7)
Current infection		13 (6.1)
Seropositive inactive		78 (36.3)
Active HCV infection, n (%)		6 (2.8)
AIDS onset, n (%)		23 (10.7)
Treatment regimen, n (%)		
DTG + (TDF/FTC or TAF/FTC)		113 (52.6)
DTG/ABC/3TC		59 (27.4)
RAL + (TDF/FTC or TAF/FTC)		20 (9.3)
EVG/cobi/TAF/FTC		17 (7.9)
Others		6 (2.8)
HIV-RNA viral load, copies/mL, median (IQR)		62,400 (18,500–151,500)
CD4^+^ cell count, cells/µL, median (IQR)		314 (133–455.5)
eGFR, mL/min/1.73 m^2^, median (IQR)		90.9 (78.2–102.5)

*SD* Standard deviation, *DTG + (TDF/FTC or TAF/FTC)* Dolutegravir + (tenofovir disoproxil fumarate/emtricitabine or tenofovir alafenamide fumarate/emtricitabine), *DTG/ABC/3TC* Dolutegravir/abacavir/lamivudine, *RAL + (TDF/FTC or TAF/FTC)* Raltegravir + (tenofovir disoproxil fumarate/emtricitabine or tenofovir alafenamide fumarate/emtricitabine), *EVG/cobi/TAF/FTC* Elvitegravir/cobicistat/ tenofovir alafenamide fumarate/emtricitabine, *eGFR* Estimated glomerular filtration rate, *HBV* Hepatitis B virus, *HCV* Hepatitis C virus, *IQR* Interquartile range

### Adherence and virological outcomes

Data were available at 4, 24, and 48 weeks for 207 (96.3%), 188 (87.4%), and 177 (82.3%) participants, respectively. The mean adherence rates at 4, 24, and 48 weeks were 99.2% (SE, 0.2%), 98.4% (SE, 0.4%), and 96.0% (SE, 1.2%), respectively. Adherence rates less than 100% were observed for 11.1%, 30.9%, and 28.3% of participants at 4, 24, and 48 weeks, respectively (Table 2). The reasons for missing ART doses at 48 weeks were as follows: “forgot” (68.0%); “pill not with participant” (18.0%); and “too busy to take them” (12.0%). The reason “slept through dose” was reported by 17.4% at 4 weeks; however, this rate decreased to 4.0% at 48 weeks. The virological outcome of virological success (HIV-RNA level < 50 copies/mL) was achieved by 180 (83.7%) at 48 weeks. However, 25 (11.6%) missed their tests because of time deviation or hospital transfer.

**Table 2:** Adherence to anti-retroviral therapy and reasons for missed medication

	Week 4 (*n* = 207)	Week 24 (*n* = 188)	Week 48 (*n* = 177)
Mean (SE)	99.2 (0.2)	98.4 (0.4)	96.0 (1.2)
95% CI	98.8–99.6	97.6–99.2	93.7–98.3
Range	80–100	37–100	0–100
≥ 95%, n (%)	195 (90.7%)	177 (82.3%)	160 (74.4%)
< 100, n (%)	23 (11.1%)	58 (30.9%)	50 (28.3%)
Reasons for missed medications among VAS < 100%, n (%)			
Forgot	12 (52.2%)	31 (63.8%)	34 (68.0%)
Pill not with participant	6 (26.1%)	8 (13.8%)	9 (18.0%)
Too busy to take them	2 (8.7%)	10 (17.2%)	6 (12.0%)
Too many pills to take	0 (0%)	0 (0%)	0 (0%)
Medications make me sick	1 (4.4%)	0 (0%)	0 (0%)
Did not want others to see me take pills	2 (8.7%)	3 (5.2%)	1 (2.0%)
Slept through dose	4 (17.4%)	17 (29.3%)	2 (4.0%)
Felt depressed or overwhelmed	0 (0%)	0 (0%)	0 (0%)
Could not follow the directions	0 (0%)	0 (0%)	0 (0%)
Too drunk or high	0 (0%)	1 (1.7%)	1 (2.0%)
Traveling	1 (4.4%)	2 (3.5%)	0 (0%)
Ran out of medications	1 (4.4%)	12 (1.7%)	2 (4.0%)
Others	0 (0%)	4 (6.9%)	5 (10.0%)

*SE* Standard error, *CI* Confidence interval, *VAS* Visual analogue scale

### Decisional conflict and HRQL

The mean DCS total score was 27.3 (SD, 13.5). Fifty (23.3%) participants had a score ≥ 37.5, 82 (38.1%) had a score of 25 to 37.5, and 79 (36.7%) had a score ≤ 25. Among the subscores, those for uncertainty, informed, values clarity, and effective decision were normal (range, 25–37.5), and those for support were low (19.6). The MOS-HIV scores at baseline and 4, 24, and 48 weeks are shown in Table 3. The mean MOS-HIV scores at 48 weeks were as follows: general health perceptions, 66.9 (SE, 1.4); pain, 66.3 (SE, 1.8); physical functioning, 94.3 (SE, 0.8); role functioning, 90.6 (SE, 2.0); social functioning, 90.7 (SE, 1.2); energy/fatigue, 46.1 (SE, 0.8); mental health, 72.2 (SE, 1.5); health distress, 76.0 (SE, 1.7); cognitive function, 78.2 (SE, 1.4); quality of life, 61.1 (SE, 1.5); and health transition, 61.1 (SE, 1.5). Overall, the MOS-HIV score tended to increase after ART. However, the energy/fatigue score remained unchanged, and the pain score increased at 4 and 24 weeks but was below baseline at 48 weeks.

**Table 3:** Changes in MOS-HIV subscale scores over time

	Baseline (*n* = 207)	Week 4 (*n* = 207)	Week 24 (*n* = 188)	Week 48 (*n* = 177)
General health perceptions	53.3 (1.5)	59.5 (1.4)	66.2 (1.3)	66.9 (1.4)
Pain	71.9 (1.9)	82.4 (1.4)	82.1 (1.6)	66.3 (1.8)
Physical functioning	90.7 (1.0)	93.1 (0.8)	94.1 (0.9)	94.3 (0.8)
Role functioning	79.5 (2.5)	87.3 (2.1)	89.8 (2.0)	90.6 (2.0)
Social functioning	83.4 (1.6)	87.0 (1.3)	89.3 (1.2)	90.7 (1.2)
Energy / Fatigue	47.2 (0.9)	47.0 (0.9)	45.2 (0.9)	46.1 (0.8)
Mental health	63.2 (1.5)	69.9 (1.3)	73.5 (1.4)	72.2 (1.5)
Health distress	62.6 (1.9)	72.9 (1.7)	76.0 (1.6)	76.0 (1.7)
Cognitive function	72.8 (1.5)	78.8 (1.3)	77.7 (1.4)	78.2 (1.4)
Quality of life	46.5 (1.5)	55.3 (1.5)	59.9 (1.4)	61.1 (1.5)
Health transition	54.6 (1.5)	60.2 (1.3)	57.9 (1.3)	61.1 (1.5)

Data are expressed as the mean (standard error)

*MOS-HIV* Medical Outcomes Study Human Immunodeficiency Virus Health Survey

### Relationships among adherence, decisional conflict, and HRQL

**Fig. 2:**
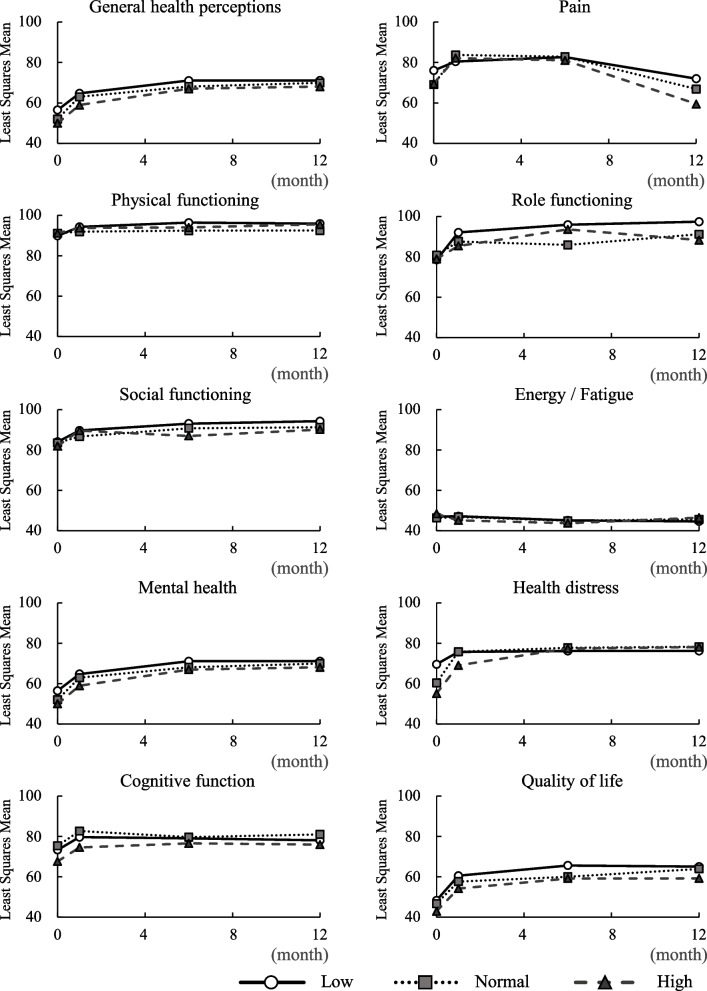
Longitudinal changes in MOS-HIV subscores according to the DCS score level (low, normal, high) after anti-retroviral therapy. MOS-HIV, Medical Outcomes Study Human Immunodeficiency Virus Health Survey; DCS, decisional conflict scale

Because medication adherence was high, we could not examine the relationships between adherence and other factors, decisional conflict, and HRQL. The least squares mean of the longitudinal MOS-HIV subscores adjusted for the baseline MOS-HIV score, AIDS history, and CD4 counts, according to the DCS score level (high, normal, low) at ART initiation, are shown in Fig. 2. There was no interaction between DCS score level and timepoints. The results of the trend test showed a tendency for the group with higher DCS scores to have lower general health perception (*p* = 0.025) and quality of life (*p* = 0.018) and possibly, lower role functioning (*p* = 0.0512), social functioning (*p* = 0.081), and cognitive function (*p* = 0.086). The differences in the least squares means of the MOS-HIV subscores adjusted for baseline MOS-HIV score, AIDS history, and CD4 count according to the DCS score level (high, normal, low) at the timepoints of ART initiation are shown in Table 4. Regardless of the DCS score level, the general health perception increased from week 4 up to week 24 and week 48; on the other hand, the role functioning and energy/fatigue at all time points showed no differences regardless of the DCS score level. Regardless of the DCS score level, the pain at week 48 decreased compared to that in week 4 and week 24. The least squares mean of the longitudinal MOS-HIV subscores adjusted for baseline MOS-HIV score, AIDS history, CD4, and timepoints according to the DCS score level for the entire study period are shown in Table 5. The MOS-HIV subscores of the low and high DCS score groups differed significantly for general health perception (69.2 vs. 64.4; difference: -4.8; 95% CI: -9.0 to -6.2; *p* = 0.025) and quality of life (64.0 vs. 57.7; difference: -6.3; 95% CI: -11.5 to -1.1; *p* = 0.018). Similarly, there was a possibility of difference in social functioning (92.4 vs. 88.8; difference: -3.6; 95% CI: -7.7 to 0.4; *p* = 0.081) and cognitive function (79.0 vs. 75.3; difference: -3.7; 95% CI: -8.0 to 0.5; *p* = 0.086). Role functioning showed a significant difference between the low and normal DCS score groups (95.1 vs. 88.5; difference: -6.6; 95% CI: -13.0 to -0.3; *p* = 0.041) and a trend towards a difference between the low and high DCS score groups; however, this difference was not significant (95.1 vs. 88.7; difference: -6.4; 95% CI: -12.9 to 0.0; *p* = 0.051).

**Table 4:** MOS-HIV subscale score differences (LSM) by DCS score level at timepoints after antiretroviral therapy

	DCS	Week 4 vs Week 24		Week 24 vs Week 48		Week 4 vs Week 48	
		Difference (SD)	*P*-value	Difference (SD)	*P*-value	Difference (SD)	*P*-value
Global health perception	Low	-6.29 (2.12)	0.0033	-0.05 (1.54)	0.9742	-6.34 (2.06)	0.0024
	Normal	-5.09 (1.59)	0.0016	-1.89 (1.49)	0.2065	-6.98 (2.19)	0.0017
	High	-8.05 (2.23)	0.0004	-1.03 (1.88)	0.584	-9.08 (2.47)	0.0004
Pain	Low	-2.19 (2.55)	0.3927	10.74 (3.55)	0.0028	8.55 (3.78)	0.0249
	Normal	0.90 (2.49)	0.7187	16.06 (3.44)	<.0001	16.96 (3.16)	<.0001
	High	1.06 (3.41)	0.7571	21.62 (3.65)	<.0001	22.68 (3.49)	<.0001
Physical functioning	Low	-2.12 (0.84)	0.0125	0.47 (0.86)	0.5812	-1.65 (0.91)	0.0715
	Normal	-0.54 (1.01)	0.5933	-0.64 (1.07)	0.5513	-0.10 (1.02)	0.9215
	High	-0.17 (1.10)	0.8767	-1.59 (1.02)	0.1185	-1.76 (1.18)	0.1371
Role functioning	Low	-3.84 (3.11)	0.2179	-1.53 (2.13)	0.4748	-5.37 (3.54)	0.1310
	Normal	1.72 (3.17)	0.5893	-5.31 (3.70)	0.1527	-3.59 (3.28)	0.2753
	High	-8.24 (5.31)	0.1221	5.35 (4.31)	0.2158	-2.89 (5.11)	0.5725
Social functioning	Low	-3.40 (1.95)	0.0829	-1.14 (1.55)	0.4656	-4.54 (1.73)	0.0092
	Normal	-4.05 (1.81)	0.0267	-0.51 (2.00)	0.7994	-4.55 (2.23)	0.0424
	High	2.59 (3.18)	0.4163	-3.25 (2.86)	0.2580	-0.65 (2.66)	0.8057
Energy / Fatigue	Low	2.04 (1.84)	0.2683	0.41 (1.43)	0.7745	2.45 (1.73)	0.1590
	Normal	1.89 (1.69)	0.2645	-0.77 (1.93)	0.6921	1.12 (1.76)	0.5244
	High	1.40 (2.00)	0.4843	-2.71 (1.89)	0.1532	-1.31 (1.74)	0.4527
Mental health	Low	-0.23 (2.25)	0.9195	0.28 (1.75)	0.8713	0.06 (2.31)	0.9803
	Normal	-3.89 (1.68)	0.0215	2.19 (1.54)	0.1562	-1.70 (1.87)	0.3637
	High	-6.61 (2.10)	0.0019	1.74 (2.02)	0.3901	-4.88 (2.18)	0.0263
Health distress	Low	-0.57 (2.18)	0.7939	0.03 (1.91)	0.9876	-0.54 (2.03)	0.7902
	Normal	-2.00 (2.22)	0.3694	-0.57 (2.43)	0.8151	-2.57 (2.87)	0.3725
	High	-8.29 (2.58)	0.0016	-0.70 (2.30)	0.7623	-8.99 (3.05)	0.0036
Cognitive function	Low	0.55 (2.22)	0.8047	0.99 (2.06)	0.6314	1.54 (2.32)	0.5078
	Normal	2.93 (1.54)	0.0589	-1.40 (1.88)	0.4591	1.54 (1.86)	0.4094
	High	-1.99 (2.98)	0.5064	0.64 (2.39)	0.7908	-1.35 (2.76)	0.6253
Quality of life	Low	-5.09 (2.52)	0.0444	0.59 (2.51)	0.8153	-4.51 (2.17)	0.0394
	Normal	-2.42 (2.06)	0.2415	-3.91 (2.05)	0.057	-6.34 (2.56)	0.0143
	High	-5.06 (3.03)	0.0972	-0.06 (2.32)	0.9801	-5.11 (3.23)	0.1154

*MOS-HIV* Medical Outcomes Study Human Immunodeficiency Virus Health Survey, *DCS* Decisional conflict scale, *LSM* Least squares mean,　*SD* Standard error

**Table 5:** MOS-HIV subscale scores according to the DCS score level after anti-retroviral therapy

	DCS			Low vs. Normal				Low vs. High			
	Low (LSM)	Normal (LSM)	High (LSM)	Difference (LSM)	95% CI		*P*-value	Difference (LSM)	95% CI		*P*-value
General health perceptions	69.2	67.0	64.4	-2.2	-6.0	1.6	0.2444	-4.8	-9.0	-6.2	0.0248
Pain	78.2	77.9	74.5	-0.2	-4.7	4.3	0.9248	-3.6	-9.0	1.7	0.1482
Physical functioning	95.3	92.4	94.6	-2.9	-5.6	-0.3	0.0304	-0.7	-3.3	1.8	0.5731
Role functioning	95.1	88.5	88.7	-6.6	-13.0	-0.3	0.0410	-6.4	-12.9	0.0	0.0512
Social functioning	92.4	89.7	88.8	-2.7	-6.6	-1.1	0.0839	-3.6	-7.7	0.4	0.0805
Energy / Fatigue	45.7	45.9	45.2	0.2	-2.3	2.7	0.8772	-0.4	-3.3	2.4	0.7692
Mental health	72.3	72.9	71.8	0.6	-3.4	4.5	0.7802	0.5	-5.0	4.0	0.8200
Health distress	76.6	77.5	73.9	0.9	-3.4	5.3	0.6685	-2.7	-8.5	3.1	0.3637
Cognitive function	79.0	81.4	75.3	2.5	-1.0	5.9	0.1578	-3.7	-8.0	0.5	0.0855
Quality of life	64.0	60.3	57.7	-3.7	-8.4	1.0	0.1261	-6.3	-11.5	-1.1	0.0181

*MOS-HIV* Medical Outcomes Study Human Immunodeficiency Virus Health Survey, *DCS* Decisional conflict scale, *LSM* Least squares mean, *CI* Confidence interval

## Discussion

This study investigated medication adherence by treatment-naïve people living with HIV and examined the decisional conflicts and HRQL that affect adherence. The mean level of adherence was maintained at a high level (> 95%). Decisional conflict ranged from low to high, and high conflicts tended to be associated with low HRQL.

A meta-analysis of adherence and virological failure suggested that even low adherence levels of 80–90% were sufficient for viral suppression; however, an adherence level of > 95% should be the target [5]. For treatment efficacy, the success of virological treatment, which was the most commonly used treatment by the participants in this study, after 48 weeks of a clinical study of dolutegravir (SPRING-2) was 88% [20]. In the present study, the mean adherence rate at the end of 48 weeks of ART was 96.0%, with 74.4% and 71.7% reporting adherence rates of ≥ 95% and 100%, respectively; the virological success rate was 83.7%. Treatment-naïve people living with HIV in Japan exhibited the high adherence required for viral suppression, and virological success was comparable to that reported by the SPRING-2 study. Virological success rates may have been underestimated because of factors such as missed time, hospital transfers, and study discontinuation. There have been no reports of the follow-up of subjective adherence and virological outcomes in Japan; therefore, this study provides new insights.

According to previous studies, factors associated with high adherence may include simple regimens (fewer doses, single-tablet regimens) [21–24], history of opportunistic diseases [25], and good communication with health care providers [8, 9]. However, factors associated with non-adherence may include low literacy levels [7, 8], medication side effects [26, 27], mental health issues such as depression [27–30], and stigma [31, 32]. Factors influencing adherence could not be tested because of the small sample size of non-adherent participants in the present study; however, it is considered that the simpler regimen contributed to good adherence. In Japan, multidisciplinary interventions are recommended to improve adherence. All facilities involved in this study were AIDS care hospitals providing multidisciplinary support systems for people living with HIV. However, the DCS support score was low. The reasons for missed doses—“forgot” and “too busy to take them”—were the same as those reported by a previous study [7] but “slept through dose” and “pill not with the participant” could have been prevented by the education provided. This suggests that the support system for people living with HIV needs to be further enhanced.

In a previous study, people living with HIV improved their HRQL with the early initiation of ART, even if they were asymptomatic at the start of treatment when adherence was good [19, 33]; however, it declined with side effects [34, 35]. Most of the MOS-HIV subscores in this study were improved at 4 weeks; however, at 24 and 48 weeks, they were almost equal and were considered to have reached a plateau. The improvement in HRQL may be attributed to the improvement in HIV-related symptoms with ART, minor side effects of ART, and good adherence. In a meta-analysis comparing ART-treated and non-treated groups, the impact of ART on the HRQL was limited, with diagnosis time and hospital services being significantly associated with improved HRQL [36]. Furthermore, people living with HIV are expected to experience significant changes in HRQL during long-term treatment [37]. Therefore, continuous support is necessary, and it may be useful to check adherence as an indicator of HRQL. Notably, there was no improvement in energy/fatigue or pain scores with ART in this study. HIV-related fatigue has been reported by 33–88% of patients and was highly associated with unemployment and inadequate income, ART, sleep disturbances, depression, and anxiety; however, no association between the CD4 count and HIV viral load was observed [38]. ART reportedly improves energy/fatigue [39], but the patient-reported HIV symptom index showed the appearance of fatigue [40]. People living with HIV are at higher risk for chronic pain, including musculoskeletal pain, painful neuropathy, and headaches. Chronic pain is also associated with cognitive impairment and mental health [41–43]. In this study, there was no improvement in energy/fatigue and pain despite improvements in physical functioning and mental health with ART. Factors such as unstudied ART adverse events, cognitive function, and stigma may have influenced the results. Validation using more specific measures of energy/fatigue and pain is warranted.

Decisional conflict experienced by cancer patients is correlated with the HRQL, and more knowledge of treatment and side effects is associated with improved HRQL after treatment [44, 45]. When the MOS-HIV subscores were adjusted for CD4 and a history of AIDS, the group with high decisional conflict had predominantly lower general health perceptions and quality of life subscores than the group with low decisional conflict. Although the HRQL of people living with HIV is influenced by the CD4 count and opportunistic infections [13, 46], it was more influenced by decisional conflict in the present study. Similar to cancer patients, decisional conflicts experienced by people living with HIV may be related to their HRQL. Because the DCS subscore for “informed” was normal, information about therapeutic drugs was provided; this might have contributed to the HRQL. In the low DCS group, role functioning tended to differ between the normal and high DCS scores. Role functioning indicates the influence of health status on work and school attendance and may be influenced by AIDS and CD4 counts. However, there are no reports demonstrating a relationship between role functioning and DCS; this is an important research topic for future studies. Multidisciplinary counseling is considered an effective approach to reducing decisional conflict [10]. Information and counseling provided by multidisciplinary professionals at the beginning of treatment may reduce decisional conflict and improve the HRQL.

The limitations of this study included selection bias and information bias. Selection bias is caused by factors influencing the individual’s decision regarding whether to participate in a study. This study was an observational study based on individual reports using electronic media. Some eligible patients did not agree to participate in the study because of their lack of continuous medical visits, severe cognitive impairment, or difficulty using electronic media. This indicates the possibility that participants with good adherence were selected for the study. The present study was conducted according to the hypothesis of “increased adherence and better HRQL leading to decreased decisional conflict.“ However, because of good adherence, this hypothesis could not be tested. The results of this study may be limited to the group with good adherence. Regarding information bias, this study may have over-reported adherence because it was a self-reported adherence survey conducted mainly by medical personnel. Self-report adherence surveys have been shown to have a tendency to overestimate results [47]. According to a web-based survey conducted by non-medical HIV researchers and parties in Japan, 56.4% of the respondents reported never forgetting to take their medications for a month [48]. This result deviates from that of the present study, and these biases may have occurred. Additionally, this study did not collect data regarding factors such as depression, psychiatric disorders, sexual behavior, or social stigma, which may be related to adherence and the HRQL. Therefore, these points should be considered when generalizing the results of the present study.

## Conclusion

Adherence among people living with HIV was maintained at a high level, and ART tended to improve the HRQL. Additionally, participants with high levels of decisional conflict at the time of treatment initiation may have poorer HRQL for up to 48 weeks compared to those with low levels of decisional conflict. In other words, reducing conflict at treatment initiation may lead to improved HRQL. Therefore, it is important to enhance social support, such as nursing care and welfare by policymakers, support groups for people living with HIV, support from local communities, and medical support from multidisciplinary teams.

## Data Availability

Due to the nature of the study, participants did not agree to having their data to be shared publicly, so supporting data is unavailable.

## Abbreviations

HIV: Human immunodeficiency virus
AIDS: Acquired immunodeficiency syndrome
ART: Anti-retroviral therapy
HRQL: Health-related quality of life
VAS: Visual analogue scale
DCS: Decisional conflict scale
MOS-HIV: Medical outcomes study- HIV
